# Exploring the association between sleep duration and cancer risk in middle-aged and older Chinese adults: observations from a representative cohort study (2011–2020)

**DOI:** 10.1186/s12889-024-19313-z

**Published:** 2024-07-08

**Authors:** Yang Jiang, Xinyue Gu, Xiao Yang, Aidi Sun, Huixin Sun

**Affiliations:** 1 Department of Pathology, Harbin Medical University Cancer Hospital, Harbin City, 150081 People’s Republic of China; 2 Colorectal Surgery, Harbin Medical University Cancer Hospital, Harbin City, 150081 People’s Republic of China; 3 Department of Environmental Health, The Center for Disease Prevention and Control of Nangang district, 150040 Harbin City, People’s Republic of China; 4 Operating Room, Harbin Medical University Cancer Hospital, Baojian Road, Harbin City, 150081 People’s Republic of China; 5 Institute of cancer prevention and treatment, Harbin Medical University, 157th Baojian Road, Nangang district, Harbin City, 150081 People’s Republic of China

**Keywords:** Sleep duration, Cancer incidence, Middle-aged and elderly, Aging, Chinese middle aged and elderly

## Abstract

**Background:**

This prospective cohort study aimed to investigate the relationship between sleep duration and cancer incidence among 9996 participants over a median follow-up period of 9 years.

**Methods:**

Participants without cancer at baseline were followed for over 88,790 person-years. The incidence of cancer and sleep duration was self-reported. The relationship between sleep duration and cancer incidence was analyzed using Cox proportional hazards models adjusted for various confounding factors, including age, gender, lifestyle factors, and comorbidities.

**Results:**

During the follow-up, 325 participants were diagnosed with incident cancer, resulting in an incidence rate of 20.49 per 1000 person-years. After adjusting for confounders, a total sleep duration of less than 6 h was significantly associated with an increased risk of cancer (HR: 1.27; 95% CI: 1.01–1.61). This association was particularly strong for cancers in the digestive and respiratory systems (HR: 1.41; 95% CI: 1.03–1.93). Longer sleep durations (> 9 h) showed a potential increase in cancer risk, but results were not consistently significant. Age-stratified analyses revealed a similar significant increase in cancer incidence among individuals aged 60 years or younger with less than 6 h of sleep per day, showing a 35% increase in overall cancer risk and an 83% increase in digestive and respiratory system cancer. No significant association was found between nocturnal sleep durations or daytime naps and cancer incidence. However, a significant interaction was observed between daytime naps longer than 30 min and cancer incidence in women (*p* = 0.041).

**Conclusions:**

We observed that short sleep duration may increase the risk of cancer, particularly cancers in the digestive and respiratory systems. Additionally, while longer sleep durations might also increase cancer risk, this finding requires validation with larger sample sizes.

**Supplementary Information:**

The online version contains supplementary material available at 10.1186/s12889-024-19313-z.

## Background

Cancer is one of the leading causes of death and a main economic burden in China, with an increasing prevalence and significant health hazards [1]. In 2020, there were an estimated 19.3 million new cancer cases worldwide (18.1 million excluding nonmelanoma skin cancer) and nearly 10.0 million cancer-related deaths (9.9 million excluding nonmelanoma skin cancer) [2].Numerous factors contribute to the development and progression of cancer, including genetic predisposition, environmental exposures, lifestyle choices, and comorbid conditions [3–6].

Among the various lifestyle factors that have been implicated in cancer risk, sleep has emerged as a critical yet understudied determinant [7–9]. Sleep is essential for maintaining physiological homeostasis and overall well-being, playing a crucial role in various biological processes, including immune function, hormonal regulation, and cellular repair [10, 11]. Regrettably, inadequate sleep is a common issue in today’s society, with over one-third of adults in the Americas, Europe, and Asia getting less than the recommended 7 h of sleep per night as advised by public health authorities for maintaining good health [12–15]. Disruptions in sleep patterns have been associated with an increased risk of several chronic diseases [16], including cancer [17]. Consequently, researchers have carried out comprehensive investigations on sleep disorders, with a major emphasis on rapid eye movement (REM) sleep behavior disorders or those induced by conditions like obstructive sleep apnea (OSA) [18]. The length of sleep is linked to the development and outcome of different illnesses [19, 20].

A systematic review summarized 21 published studies on the relationship between sleep and cancer risk incidence, noting the absence of cohort studies from China [21]. However, recent research from the Kailuan cohort conducted in Tangshan, China, reported that sleep duration trajectories and quality are closely associated with cancer risk [8].Despite growing recognition of the importance of sleep in cancer etiology, current research on the relationship between sleep duration and cancer risk remains limited and inconclusive in China. There have been relatively few studies [22–24] on the relationship between sleep duration and cancer risk in previous research, especially lacking long-term cohort studies with large nationally representative samples. Existing studies have predominantly focused on specific cancer types or have been retrospective in nature [25, 26], hindering our understanding of the broader impact of sleep on cancer incidence.

In this study, we aim to address this gap by conducting a cohort study using data from the China Health and Retirement Longitudinal Study (CHARLS) database covering the period from 2011 to 2020. By analyzing the sleep patterns and cancer incidence risk among a representative sample of Chinese middle-age and older adults, we seek to examine the hypothesis that sleep duration plays a significant role in influencing the onset of cancer. This research has the potential to provide valuable insights into the relationship between sleep behavior and cancer risk, contributing to the development of preventive strategies and interventions in cancer management.

## Methods

## Study design

This study utilized a longitudinal cohort study design to investigate the relationship between sleep duration and cancer incidence. Data collected from the CHARLS database from the years 2011 to 2020. CHARLS is a nationally representative survey of the Chinese population aged 45 and older [27]. It collects data on a wide range of topics including health, retirement, income, and social support. The database has been collected every two years since 2011, and involves home interviews, medical examinations, and other data collection activities. Information on health, demographics, socioeconomic status, and physical and physiological measurements was gathered from 28 provinces and 150 counties across China. More information about CHARLS can be found on the official website (https://charls.pku.edu.cn/) or in publications [28].

### Inclusion and exclusion criteria

A total of 17,705 participants were enrolled in the 2011 baseline, with 10,257 eligible participants retained after excluding missing information on cancer diagnosis and participants diagnosed with cancer at baseline, and excluding those with missing data on sleep duration and aged under 45. Considering that cancer diagnoses in this study were self-reported by participants, potential underreporting may exist. Given evidence suggesting an association between cancer and renal function impairment [29], and considering the relationship between sleep and renal function [29], patients with renal function impairment were also excluded. Further details can be seen in Figure S1.

### Variable definition

#### Exposure

Subjective sleep duration was measured retrospectively for the month before the survey was conducted. Participants were asked by trained stuff by the question” During the past month, how many hours of actual sleep did you get at night (average hours for one night)?”and “During the past month, how long did you take a nap after lunch?”. Daytime sleep duration was categorized as 0 min/day, ≤ 30 min/day and over 30 min/day. Nocturnal sleep duration was categorized as extremely short (≤ 6 h/night), short (6–9 h/night) and long (> 9 h/night) [30]. The average total daily sleep duration (the sum of daytime and nocturnal sleep duration) was divided into short (≤ 6 h per day), moderate (6–9 h per day) and long (> 9 h per day).

#### Main outcome measures

The outcome of the study is cancer (excluding non-melanoma skin cancer). Participants reported their cancer incidences. Based on the location of cancer occurrence, cancers were categorized into corresponding systems. Due to limitations in the number of cancer cases, we ultimately grouped cancer types into the digestive and respiratory systems (digestive system, including cancers occurring in the oral cavity, oesophagus, stomach, liver, pancreas, colon or rectum; respiratory system, including cancers occurring in the larynx, other pharynx, and lung) and other systems (including cancers of the brain, thyroid, breast, kidney, ovary, cervix, endometrium, bladder, skin, non-Hodgkin lymphoma, leukemia, and other organs). Participants were followed from baseline until cancer was detected during the survey or until the last available survey prior to 2020, whichever came first. We calculated person-years for each individual as the number of years from baseline to the first diagnosis of cancer, death, censoring, or the end of follow-up.

#### Covariates

Age was calculated by 2011 deducted their birth year. Height and weight measurements of individuals were taken without shoes and in light clothing using a calibrated weight scale and stadiometer equipment. Height was accurately measured to the nearest 0.1 cm, and weight was measured to the nearest 0.1 kg. [31]. According to the WHO guidelines for Chinese individuals, overweight and obesity were defined as having a body mass index (BMI) of 23.0 and 27.5 or higher, respectively [32]. Participants’ waist circumference was measured after exhalation using a soft measuring tape around their waist at the navel level. Abdominal obesity was defined as WC ≥ 90 cm for males and ≥ 80 cm for females, based on the International Diabetes Federation cut-offs for the Chinese population. [33].

The highest education levels were categorized as illiterate (no formal education), primary (elementary school), secondary (middle or high school), and college or higher (two-/three-year college and higher degrees). Participants were classified as smokers if they had smoked at least 100 cigarettes in their lifetime, with smoking status categorized as never, current, or former smokers. Drinking was defined as consuming alcohol at least once a month, with drinking history categorized as current, never, or former drinkers. Type 2 diabetes was defined as either having a fasting plasma glucose level above 126 mg/dL or currently using anti-diabetic medications. [34]. Participants’ blood pressure (BP) was determined by averaging three measurements. Hypertension was defined as a diastolic BP ≥ 90 and/or systolic BP ≥ 140 mmHg, or self-reported use of medications for hypertension treatment. We divided the participants into 2 groups (rural and Urban Community). Dyslipidemia is characterized by elevated levels of total cholesterol (TC), triglycerides (TG), and low-density lipoprotein cholesterol (LDL-C), as well as decreased levels of high-density lipoprotein cholesterol (HDL-C). Dyslipidemia can be identified by phenotypes such as TC > 6.2 mmol/L, TG ≥ 2.3 mmol/L, LDL-C ≥ 4.1 mmol/L, and HDL-C < 1.0 mmol/L in males or < 1.3 mmol/L in females, or self-reported dyslipidemia [35, 36].

According to the Chronic Kidney Disease-Epidemiology Collaboration equation, glomerular filtration rate (GFR) was calculated [37]: In men, ① serum creatinine ≤ 0.9 mg/dl: GFR = 141 * (serum creatinine (mg/dl)/0.7)-0.441 * (0.993)*age; ② serum creatinine > 0.9 mg/dl; GFR = 144* (serum creatinine (mg/dl)/0.7)-0.441 * (0.993)*age. In women, ① serum creatinine ≤ 0.7 mg/dl: GFR = 144 * (serum creatinine (mg/dl)/0.7)-0.329 *(0.993)*age; ② serum creatinine > 0.7 mg/dl: GFR = 144 /* (serum creatinine (mg/dl)/0.7)-1.209* (0.993)*age. An eGFR of less than 80 mL/min/1.73 m² was considered indicative of renal impairment [37].

### Data analysis

Missing data distribution plots were used to evaluate the distribution of missing data. The Multiple Imputation by Chained Equations (MICE) method was applied to impute the missing data. The percentage of missing cases is detailed in Table S1.

Baseline characteristics were summarized by subgroups based on categories of total sleep duration and evaluated using the Mann–Whitney U or Chi-square tests. The relationship between sleep duration and cancer was analyzed using Cox proportional hazards regression models with competing risk analysis, ensuring that the proportional hazard assumption was met. Death was treated as a competing event, and the date of death was obtained from the reports of participants’ relatives or friends. Adjusted hazard ratios (HRs) with 95% confidence intervals (CIs) were calculated for the incidence of cancer [38]. The models were adjusted to account for the competing risk of death.

We fitted a crude univariable model (model 1) and two multivariable models (model 2 and model 3). Model 2 accounted for confounding factors such as sex, age (continuous), education, smoking, drinking, and residence. Model 3 further included adjustments for hypertension, diabetes, dyslipidemia, waist circumference, and BMI.

Subgroup analyses by gender (male vs. female) and age (≤ 60 vs. >60 years) were conducted. Additionally, interaction analyses for the subgroups were performed. Sensitivity analyses included handling missing data and excluding cancer cases that occurred within the first two years of follow-up.

All reported p-values are two-tailed, with significance defined as *p* < 0.05. The analyses were conducted using R 4.2.3 (R-project, Vienna, Austria).

### Ethical considerations

All participants signed informed consent. The data obtained in this study were approved by the Peking University/Institutional Review Board (household survey: IRB00001052-11015; biomarker: IRB00001052-11014).

## Results

The study included 9996 participants without cancer at baseline. Participants were followed for over 88,790 person-years (median, 9 years) in total. We observed that 325 participants developed incident cancer (incidence rate: 200.49 per 10,000 person-years). Totally, 98 participants lost to follow-up and 31 were died for unknown reason, the follow-up rate was 99.0%.

Table 1 displays the baseline characteristics of participants based on their total sleep duration. Those who slept shorter tended to be older, female, and have higher rates of rural residency and normal weight. They also had a lower prevalence of current smoking and drinking, and a lower level of advanced education. In contrast, those who slept longer had higher rates of current smoking and drinking, advanced education, cardiovascular disease, and slightly higher median values for waist circumference, height, weight, and BMI.

**Table 1:** Baseline characteristics of participants

Characteristic	Overall, *N* = 9,996	Day time sleep duration(<6 h), *N* = 3,557	Day time sleep duration(6–9 h), *N* = 5,420	Day time sleep duration(>9 h), *N* = 1,019
Gender, *n* (%)				
Female	5,339.0 (53.4%)	2,087.0 (58.7%)	2,751.0 (50.8%)	501.0 (49.2%)
Male	4,657.0 (46.6%)	1,470.0 (41.3%)	2,669.0 (49.2%)	518.0 (50.8%)
**Age, Median (IQR)**	57.00 (50.00, 63.00)	58.00 (52.00, 64.00)	56.00 (49.00, 62.00)	57.00 (50.00, 64.00)
**Smoking status, n (%)**				
Currently smoking	3,078.0 (30.8%)	1,028.0 (28.9%)	1,719.0 (31.7%)	331.0 (32.5%)
Ever smoked	737.0 (7.4%)	247.0 (6.9%)	396.0 (7.3%)	94.0 (9.2%)
Never smoked	6,181.0 (61.8%)	2,282.0 (64.2%)	3,305.0 (61.0%)	594.0 (58.3%)
**Drinking status, n (%)**				
Currently drinking	2,608.0 (26.1%)	874.0 (24.6%)	1,448.0 (26.7%)	286.0 (28.1%)
Ever drunk	423.0 (4.2%)	173.0 (4.9%)	208.0 (3.8%)	42.0 (4.1%)
Never drink	6,965.0 (69.7%)	2,510.0 (70.6%)	3,764.0 (69.4%)	691.0 (67.8%)
**Education level, n (%)**				
Advanced education	3,207.0 (32.1%)	920.0 (25.9%)	1,959.0 (36.1%)	328.0 (32.2%)
College and above	196.0 (2.0%)	37.0 (1.0%)	148.0 (2.7%)	11.0 (1.1%)
Illiterate	2,619.0 (26.2%)	1,132.0 (31.8%)	1,201.0 (22.2%)	286.0 (28.1%)
Primary school	3,974.0 (39.8%)	1,468.0 (41.3%)	2,112.0 (39.0%)	394.0 (38.7%)
**CVD, n (%)**				
Yes	8,858.0 (88.6%)	3,083.0 (86.7%)	4,854.0 (89.6%)	921.0 (90.4%)
No	1,138.0 (11.4%)	474.0 (13.3%)	566.0 (10.4%)	98.0 (9.6%)
**eGFR, Median (IQR)**	97.91 (90.94, 104.09)	97.38 (90.26, 103.49)	98.25 (91.41, 104.38)	98.21 (91.33, 104.16)
**Hypertension, n (%)**	3,522.0 (35.2%)	1,273.0 (35.8%)	1,884.0 (34.8%)	365.0 (35.8%)
**Diabetes, n (%)**	553.0 (5.5%)	191.0 (5.4%)	304.0 (5.6%)	58.0 (5.7%)
**Dyslipidemia, n (%)**	621.0 (6.2%)	202.0 (5.7%)	357.0 (6.6%)	62.0 (6.1%)
**Residence status, n (%)**				
Rural Village	6,370.0 (63.7%)	2,350.0 (66.1%)	3,334.0 (61.5%)	686.0 (67.3%)
Urban Community	3,626.0 (36.3%)	1,207.0 (33.9%)	2,086.0 (38.5%)	333.0 (32.7%)
**Obesity status, n (%)**				
Normal weight	5,279.0 (52.8%)	1,952.0 (54.9%)	2,786.0 (51.4%)	541.0 (53.1%)
Over weight and obeisity	4,122.0 (41.2%)	1,352.0 (38.0%)	2,346.0 (43.3%)	424.0 (41.6%)
Under weight	595.0 (6.0%)	253.0 (7.1%)	288.0 (5.3%)	54.0 (5.3%)
**Nocturnal sleep duration, Median (IQR)**	7.00 (5.00, 8.00)	5.00 (4.00, 6.00)	7.00 (7.00, 8.00)	9.00 (8.00, 10.00)
**Day time sleep duration, Median (IQR)**	1.00 (0.00, 60.00)	0.00 (0.00, 20.00)	30.00 (0.00, 60.00)	90.00 (30.00, 120.00)
**WC, Median (IQR)**	84.30 (78.00, 91.40)	83.80 (77.20, 90.80)	85.00 (78.00, 92.00)	85.00 (78.00, 92.80)
**Height, Median (IQR)**	158.20 (151.90, 165.00)	156.70 (150.90, 163.50)	159.10 (152.60, 165.70)	159.00 (152.10, 165.60)
**Weight, Median (IQR)**	58.10 (51.70, 65.60)	56.60 (50.20, 63.80)	59.00 (52.50, 66.80)	58.80 (52.35, 66.30)
**BMI, Median (IQR)**	23.17 (20.93, 25.82)	22.86 (20.74, 25.46)	23.36 (21.06, 26.01)	23.28 (21.03, 26.05)
**Nocturnal sleep duration(Quatile), n (%)**				
<6 h	4,827.0 (48.3%)	3,557.0 (100.0%)	1,270.0 (23.4%)	0.0 (0.0%)
6–9 h	4,761.0 (47.6%)	0.0 (0.0%)	4,150.0 (76.6%)	611.0 (60.0%)
>9 h	408.0 (4.1%)	0.0 (0.0%)	0.0 (0.0%)	408.0 (40.0%)
**Day time sleep duration(Quatile), n (%)**				
0–30 min	6,424.0 (64.3%)	2,904.0 (81.6%)	3,235.0 (59.7%)	285.0 (28.0%)
>30 min	3,572.0 (35.7%)	653.0 (18.4%)	2,185.0 (40.3%)	734.0 (72.0%)

The relationship between sleep duration and cancer incidence is demonstrated in Table 2. After adjusting for age, gender, smoking, drinking, education, residence, hypertension, diabetes, dyslipidemia, waist circumference, BMI, and eGFR, it was found that a total sleep duration of less than 6 h significantly increased the risk of cancer, with HR and 95% CI of 1.27 (1.01, 1.61), and this hazard effect also found for digestive & respiratory system cancers (HR:1.41, 95%CI:1.03–1.93). Longer sleep durations (> 9 h) also show a potential increase in risk, but the results are not consistently significant across models and cancer types. In subgroup analyses stratified by age, a similar significant increase in cancer incidence was observed among individuals aged 60 years or younger, with those sleeping less than 6 h per day exhibiting a 35% increase in overall cancer risk and 83% increase of digestive and respiratory system cancer (Table 3). However, there was no significant association between nocturnal sleep durations and cancer incidence. Additionally, there was no significant association between sleeping more than 30 min per day and cancer risk compared to sleeping ≤ 30 min per day, with an HR (95%CI) of 1.13 (0.90, 1.42) (Table 2). Gender-stratified analyses did not reveal any significant associations between sleep duration and cancer incidence. However, there was a significant interaction between daytime naps longer than 30 min and cancer incidence in women (*p* = 0.041) (Table 4).

**Table 2:** Effects of sleep duration on incidence of cancer

Characteristic	*N*	Event *N*	Mod1			Mod2			Mod3		
			HR^1^	95% CI^1^	*p*-value	HR^1^	95% CI^1^	*p*-value	HR^1^	95% CI^1^	*p*-value
**Overall cancer**											
**Nocturnal sleep duration (hours)**	9,996	325									
6-9	4,827	141	—	—		—	—		—	—	
<6	4,761	168	1.18	0.94, 1.47	0.155	1.11	0.89, 1.39	0.360	1.13	0.90, 1.42	0.282
>9	408	16	1.34	0.80, 2.24	0.269	1.29	0.77, 2.17	0.336	1.28	0.76, 2.16	0.345
**Day time sleep duration(minutes)**	9,996	325									
≤30	6,424	196	—	—		—	—		^*—*^	^*—*^	
>30	3,572	129	1.19	0.95, 1.48	0.130	1.15	0.92, 1.44	0.225	1.13	0.90, 1.41	0.295
**Total sleep duration(hours)**	9,996	325									
6-9	5,420	153	—	—		—	—		—	—	
<6	3,557	131	1.31	1.04, 1.65	0.023	1.26	0.99, 1.59	0.059	1.27	1.01, 1.61	0.046
>9	1,019	41	1.44	1.02, 2.03	0.039	1.40	0.99, 1.98	0.054	1.39	0.98, 1.96	0.063
**Digestive&Respiratory_System**											
**Nocturnal sleep duration (hours)**	9,996	184									
6-9	4,761	79	—	—		—	—		—	—	
<6	4,827	97	1.21	0.90, 1.63	0.206	1.14	0.84, 1.53	0.40	1.12	0.83, 1.51	0.457
>9	408	8	1.18	0.57, 2.45	0.648	1.04	0.50, 2.16	0.913	1.04	0.50, 2.17	0.909
**Day time sleep duration(minutes)**	9,996	184									
≤30	6,424	110	—	—		—	—		—	—	
>30	3,572	74	1.21	0.90, 1.63	0.204	1.04	0.78, 1.41	0.773	1.09	0.81, 1.48	0.558
**Total sleep duration(hours)**	9,996	184									
6-9	5,420	82	—	—		—	—		—	—	
<6	3,557	79	1.47	1.08, 2.01	0.014	1.43	1.05, 1.95	0.025	1.41	1.03, 1.93	**0.032**
>9	1,019	23	1.50	0.94, 2.38	0.086	1.36	0.86, 2.17	0.193	1.36	0.85, 2.16	0.199
**Other System**											
**Nocturnal sleep duration (hours)**	9,996	140									
6-9	4,761	64	—	—		—	—		—	—	
<6	4,827	68	1.05	0.75, 1.48	0.785	1.00	0.71, 1.41	0.986	1.05	0.74, 1.48	0.782
>9	408	8	1.46	0.70, 3.05	0.312	1.51	0.72, 3.16	0.274	1.45	0.69, 3.03	0.325
**Day time sleep duration(minutes)**	9,996	140									
≤30	6,424	85	—	—		—	—		—	—	
>30	3,572	55	1.16	0.83, 1.63	0.379	1.28	0.91, 1.80	0.161	1.18	0.84, 1.67	0.345
**Total sleep duration(hours)**	9,996	140									
6-9	5,420	73	—	—		—	—		—	—	
<6	3,557	49	1.02	0.71, 1.47	0.898	0.96	0.67, 1.39	0.838	0.98	0.68, 1.42	0.927
>9	1,019	18	1.31	0.78, 2.20	0.303	1.36	0.81, 2.29	0.241	1.32	0.78, 2.21	0.299

**Table 3:** Effects of sleep duration on incidence of cancer grouped by age

Characteristic	Age<=60					Age>60					
	**N**	Event *N*	HR^1^	95% CI^1^	*p*-value	**N**	Event *N*	HR^1^	95% CI^1^	*p*-value	*p*_for interaction_
**Overall cancer**											
**Nocturnal sleep duration (hours)**	6,675	180				3,321	145				
6-9	3,352	81	—	—		1,409	60	—	—		
<6	3,054	87	1.14	0.84, 1.55	0.389	1,773	81	1.10	0.78, 1.54	0.588	0.608
>9	269	12	1.81	0.98, 3.32	0.056	139	4	0.71	0.26, 1.98	0.517	0.235
**Day time sleep duration(minutes)**	6,675	180				3,321	145				
≤30	4,339	113	—	—		2,085	83	—	—		
>30	2,336	67	1.10	0.81, 1.49	0.545	1,236	62	1.17	0.84, 1.64	0.348	0.515
**Total sleep duration(hours)**	6,675	180				3,321	145				
6-9	3,800	87	—	—		1,620	66	—	—		
<6	2,223	72	1.35	1.00, 1.85	0.049	1,334	59	1.15	0.80, 1.64	0.454	0.414
>9	652	21	1.42	0.88, 2.28	0.153	367	20	1.42	0.86, 2.35	0.174	0.989
**Digestive&Respiratory_System**											
**Nocturnal sleep duration (hours)**	6,675	92				3,321	92				
6-9	3,352	38	—	—		1,409	41	—	—		
<6	3,054	48	1.28	0.83, 1.96	0.259	1,773	49	1.10	0.78, 1.54	0.588	0.169
>9	269	6	1.91	0.81, 4.53	0.142	139	2	0.71	0.26, 1.98	0.517	0.359
**Day time sleep duration(minutes)**	6,675	92				3,321	92				
≤30	4,339	59	—	—		2,085	51	—	—		
>30	2,336	33	0.98	0.64, 1.51	0.935	1,236	41	1.17	0.84, 1.64	0.348	0.390
**Total sleep duration(hours)**	6,675	92				3,321	92				
6-9	3,800	39	—	—		1,620	43	—	—		
<6	2,223	42	1.83	1.18, 2.83	0.007	1,334	37	1.15	0.80, 1.64	0.454	0.062
>9	652	11	1.58	0.81, 3.08	0.182	367	12	1.42	0.86, 2.35	0.174	0.517
**Other System**											
**Nocturnal sleep duration (hours)**	6,675	85				3,321	55				
6-9	3,352	43	—	—		1,409	21	—	—		
<6	3,054	36	0.93	0.60, 1.46	0.761	1,773	32	0.97	0.64, 1.48	0.894	0.567
>9	269	6	1.61	0.68, 3.79	0.280	139	2	0.49	0.12, 2.05	0.330	0.400
**Day time sleep duration(minutes)**	6,675	85				3,321	55				
≤30	4,339	51	—	—		2,085	34	—	—		
>30	2,336	34	1.26	0.81, 1.95	0.309	1,236	21	1.20	0.79, 1.83	0.392	0.775
**Total sleep duration(hours)**	6,675	85				3,321	55				
6-9	3,800	49	—	—		1,620	24	—	—		
<6	2,223	26	0.83	0.51, 1.34	0.440	1,334	23	1.10	0.71, 1.72	0.670	0.378
>9	652	10	1.19	0.60, 2.35	0.618	367	8	1.22	0.64, 2.33	0.541	0.729

**Table 4:** Effects of sleep duration on incidence of cancer grouped by gender

Characteristic	Male					Female					*p*_for interaction_
	**N**	Event *N*	HR^1^	95% CI^1^	*p*-value	**N**	Event *N*	HR^1^	95% CI^1^	*p*-value	
**Overall cancer**											
**Nocturnal sleep duration (hours)**	4,657	160				5,339	165				
6-9	2,303	68	—	—		2,458	73	—	—		
<6	2,159	85	1.24	0.90, 1.71	0.195	2,668	83	1.32	0.69, 2.54	0.397	0.264
>9	195	7	1.06	0.48, 2.31	0.886	213	9	0.58	0.08, 4.46	0.604	0.674
**Day time sleep duration(minutes)**	4,657	160				5,339	165				
≤30	2,676	83	—	—		3,748	113	—	—		
>30	1,981	77	1.23	0.90, 1.68	0.203	1,591	52	1.12	0.59, 2.11	0.735	0.503
**Total sleep duration(hours)**	4,657	160				5,339	165				
6-9	2,669	77	—	—		2,751	76	—	—		
<6	1,470	61	1.32	0.94, 1.85	0.108	2,087	70	0.95	0.47, 1.90	0.879	0.467
>9	518	22	1.37	0.85, 2.21	0.191	501	19	0.80	0.27, 2.35	0.689	0.890
**Digestive&Respiratory_System**											
**Nocturnal sleep duration (hours)**	4,657	125				5,339	59				
6-9	2,303	54	—	—		2,458	25	—	—		
<6	2,159	65	1.24	0.90, 1.71	0.195	2,668	32	1.00	0.59, 1.70	0.995	0.617
>9	195	6	1.06	0.48, 2.31	0.886	213	2	0.84	0.20, 3.58	0.817	0.801
**Day time sleep duration(minutes)**	4,657	125				5,339	59				
≤30	2,676	63	—	—		3,748	47	—	—		
>30	1,981	62	1.23	0.90, 1.68	0.203	1,591	12	0.63	0.33, 1.20	0.160	0.041
**Total sleep duration(hours)**	4,657	125				5,339	59				
6-9	2,669	57	—	—		2,751	25	—	—		
<6	1,470	50	1.32	0.94, 1.85	0.108	2,087	29	1.35	0.79, 2.33	0.275	0.815
>9	518	18	1.37	0.85, 2.21	0.191	501	5	1.02	0.39, 2.68	0.967	0.495
**Other System**											
**Nocturnal sleep duration (hours)**	4,657	39				5,339	101				
6-9	2,303	16	—	—		2,458	48	—	—		
<6	2,159	22	1.32	0.69, 2.54	0.397	2,668	46	1.00	0.59, 1.70	0.995	0.199
>9	195	1	0.58	0.08, 4.46	0.604	213	7	0.84	0.20, 3.58	0.817	0.438
**Day time sleep duration(minutes)**	4,657	39				5,339	101				
≤30	2,676	21	—	—		3,748	64	—	—		
>30	1,981	18	1.12	0.59, 2.11	0.735	1,591	37	0.63	0.33, 1.20	0.160	0.810
**Total sleep duration(hours)**	4,657	39				5,339	101				
6-9	2,669	22	—	—		2,751	51	—	—		
<6	1,470	13	0.95	0.47, 1.90	0.879	2,087	36	1.35	0.79, 2.33	0.275	0.756
>9	518	4	0.80	0.27, 2.35	0.689	501	14	1.02	0.39, 2.68	0.967	0.416

Reanalysis of the study results using missing data, as shown in Table S2, yielded outcomes that were nearly consistent with those obtained using imputed missing values. Additionally, after excluding patients who developed cancer within the first two years of follow-up, the results remained largely consistent, as shown in Table S3. This consistency indicates the robustness of the findings.

## Discussion

The study followed nearly 10,000 participants for an average of 9 years and found that short sleep duration was associated with an increased cancer risk, particularly for cancers in the digestive and respiratory systems, compared to those with a moderate sleep duration (6–9 h per day). However, there was no significant association between longer sleep durations or napping and cancer risk.

Self-reported typical sleep characteristics and shift work were not found to be associated with pancreatic cancer risk among UK residents [25]. Similarly, no significant association was observed between sleep duration and breast cancer risk in US residents [7]. However, in Chinese residents, sleep duration trajectories and quality were found to be closely linked to cancer risk and cancer-specific mortality [8]. The relationships between sleep duration and cancer risk have been inconsistent, possibly due to variations in population and study methods. Our prospective cohort study suggests that both inadequate and excessive sleep elevate the risk of cancer. According to the recommended healthy sleep duration outlined in the Thoracic Society Statement, which suggests 6–9 h/day [39], our findings align. We found that compared to individuals who slept 6–9 h per day, those who slept less had an increased risk of cancer. Additionally, those who slept more than 9 h per day showed a nearly statistically significant increase in cancer risk. Notably, our findings align with previous research indicating a U-shaped association between sleep duration and malignant neoplasms of digestive organs [40]. Additionally, meta-analysis study indicated a J-shaped association between sleep duration and all-cause mortality, where both shortened and prolonged sleep durations were linked to increased mortality risk compared to 7 h of sleep [41]. However, the number of participants sleeping more than 9 h was very small, which might explain the lack of statistical significance in this study. This result needs to be validated by studies with larger sample sizes. While the individual hazard ratios for daytime naps longer than 30 min were not significant for either gender, the significant interaction term suggests that gender modifies the effect of daytime nap duration on cancer incidence. Further research is needed to understand the underlying mechanisms and to explore how gender-specific factors contribute to this interaction.

Moving forward, further research is warranted to elucidate the underlying mechanisms linking sleep duration to cancer risk. Investigating the biological pathways involved in sleep regulation and their potential impact on carcinogenesis could provide valuable insights. Additionally, exploring the interplay between sleep duration, lifestyle factors, and genetic predispositions may help refine risk assessment models and inform preventive strategies. In general, poor sleep quality may contribute to cancer development through various mechanisms, including reduced production of melatonin, sleep disruption, and disturbances in lifestyle [42]. Melatonin, a hormone produced by the pineal gland, plays a crucial role in regulating the circadian rhythm. Studies have suggested that shift workers, who are often exposed to light at night, may exhibit lower levels of melatonin or its metabolites, potentially influencing cancer-related processes such as proliferation, metastasis, and angiogenesis [43, 44]. Additionally, well-established risk factors for cancer, such as tobacco smoking, physical inactivity, overweight, and obesity, should be considered [45].

The strengths of our study include a large sample size and nationally representative participants. However, several limitations need to be addressed. Firstly, cancer status was self-reported, and the specific types of cancer could not be distinguished. According to estimates from the National Cancer Center (NCC) of China, approximately 4,064,000 new cancer cases were reported in 2016. This resulted in a crude incidence rate of 293.9 per 100,000 for the whole population and 1,057.56 per 100,000 for individuals aged 60 and above [46, 47]. In our study, the overall cancer incidence rate was found to be 3,251.626 per 100,000. The discrepancy in cancer incidence rates may stem from differences in the study population. Our research includes individuals aged 45 and above, while other studies might focus on different age groups. Additionally, the increasing aging population in China could contribute to variations in incidence rates. Secondly, information on sleep habits, such as the frequency of daytime naps and daytime activities, was lacking. Although older respondents consistently reported poor sleep quality, there was no standardized sleep quality questionnaire available, and sleep quality or patterns were not accounted for. Thirdly, self-reported sleep duration may have been overestimated by approximately 60 min compared to objective measurements for Chinese adults [48].

## Conclusion

Our study investigated the relationship between sleep duration and cancer incidence among 9,996 participants over a median follow-up period of 9 years. We observed a significant association between short sleep duration and the incidence of cancer, particularly cancers occurring in the digestive and respiratory systems. Although no significant associations were found between napping or nocturnal sleep and overall cancer incidence, there was an interaction between daytime napping and gender in relation to cancer risk, which warrants further investigation.

### Electronic supplementary material

Below is the link to the electronic supplementary material.

Supplementary Material 1

Supplementary Material 2

Supplementary Material 3

Supplementary Material 4

Supplementary Material 5

Supplementary Material 6

## Data Availability

The data is freely accessible on the website https://charls.pku.edu.cn/.

## Abbreviations

CHARLS: China Health and Retirement Longitudinal Study
HR: Hazard Ratio
CI: Confidence Interval
BMI: Body Mass Index
WC: Waist Circumference
BP: Blood Pressure
TC: Total Cholesterol
TG: Triglycerides
LDL-C: Low-Density Lipoprotein Cholesterol
HDL-C: High-Density Lipoprotein Cholesterol
GFR: Glomerular Filtration Rate
MICE: Multiple Imputation by Chained Equations
RCS: Restricted Cubic Splines
REM: Rapid Eye Movement
OSA: Obstructive Sleep Apnea
IRB: Institutional Review Board
