# Supplementary figures and images for: Exploring the association between sleep duration and cancer risk in middle-aged and older Chinese adults: observations from a representative cohort study (2011–2020)
Source: BMC Public Health. 2024 Jul 8;24:1819. doi: 10.1186/s12889-024-19313-z (PMC11232271; doi:10.1186/s12889-024-19313-z)

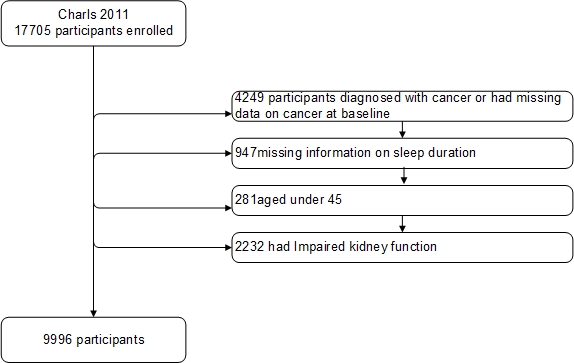

Supplement: Supplementary file 3 — Supplementary Material 3 [file 12889_2024_19313_MOESM3_ESM.jpg]
