# Supplementary material for: Exploring the association between sleep duration and cancer risk in middle-aged and older Chinese adults: observations from a representative cohort study (2011–2020)
Source: BMC Public Health. 2024 Jul 8;24:1819. doi: 10.1186/s12889-024-19313-z (PMC11232271; doi:10.1186/s12889-024-19313-z)
Supplement: Supplementary file 6 — Supplementary Material 6 [file 12889_2024_19313_MOESM6_ESM.docx]

Supplementary Materials

Table S1 Percentage of missing data

Table S2 Effects of sleep duration on incidence of cancer (with missing data)

Table S3 Effects of sleep duration on incidence of cancer (excluding patients who developed cancer within the first two years of follow-up)

Figure S1 Flow chart of participants selection

Figure S2 Distribution of missing data
